# A qualitative assessment of the perceived acceptability and feasibility of eHARTS, a mobile application for transition readiness assessment for adolescents living with HIV in South Africa

**DOI:** 10.1371/journal.pdig.0000272

**Published:** 2023-06-16

**Authors:** Messaline F. Fomo, John Newman, Thobekile Sibaya, Nompumelelo Ndlela, Sophia Hussen, Moherndran Archary, Brian C. Zanoni

**Affiliations:** 1 Hubert Department of Global Health, Rollins School of Public Health, Emory University, Atlanta, Georgia, United States of America; 2 Department of Medicine and Pediatric Infectious Diseases, Emory University, Georgia, United States of America; 3 Department of Pathology, Microbiology and Immunology, Vanderbilt University Medical Center, Nashville, Tennessee, United States of America; 4 Department of Pediatrics, Nelson Mandela School of Medicine, University of KwaZulu Natal, KwaZulu Natal, South Africa; 5 Children’s Healthcare of Atlanta, Atlanta, Georgia, United States of America; Iran University of Medical Sciences, IRAN (ISLAMIC REPUBLIC OF)

## Abstract

South Africa has the highest burden of adolescents living with HIV (ALHIV) in the world. The transition from pediatric to adult centered HIV care is a vulnerable period during which many clinical outcomes of ALHIV suffer. Transition readiness assessments may help ALHIV transition from pediatric to adult care to improve their health outcomes. Here, we evaluated the perceived acceptability and feasibility of a mobile health (mHealth) application, eHARTS, to determine transition readiness for ALHIV in South Africa. We conducted in-depth interviews with adolescents (n = 15) and healthcare providers (n = 15) at three government-supported hospitals in KwaZulu-Natal, South Africa. We used a semi-structured interview guide comprising of open-ended questions based on the unified theory of acceptance and use of technology. We did a thematic analysis of the data using an iterative, team-based coding approach to develop themes that were representative of the participants’ perspectives on the acceptability and feasibility of eHARTS. We found that most participants found eHARTS to be acceptable because of its simplicity and lack of stigma. Participants believed eHARTS was feasible as it could easily be administered within a hospital setting and integrated into regular clinic activity without disrupting patient care. Additionally, eHARTS was found to have great utility for adolescents and healthcare providers. Clinicians saw it as a valuable tool to engage adolescents and prepare them for transition. Despite concerns that eHARTS may give adolescents a wrong impression about immediate transition, participants suggested that eHARTS be framed in an empowering way as they prepare for transition to adult care. Our data showed that eHARTS is a simple, mobile transition assessment tool with perceived acceptability and feasibility for use in HIV clinics in South Africa for ALHIV. It is particularly useful for ALHIV and transitioning to adult care as it can help identify gaps in readiness for transition.

## Author summary

Adolescents living with HIV often face difficulties when transitioning from pediatric to adult HIV care. To improve their health outcomes, it is important to assess their readiness for transition. In this study, we evaluated the perceived acceptability and feasibility of eHARTS, a mobile health (mHealth) application designed to assess transition readiness for these adolescents. We interviewed both adolescents and healthcare providers at three government-supported hospitals in KwaZulu-Natal, South Africa. We found that eHARTS was well-received and practical for use in HIV clinics. It was appreciated for its simple navigation features and ability to be integrated into regular clinic activities without disrupting patient care. Our study highlights the potential of eHARTS to support clinicians in making informed judgments about an adolescent’s readiness for adult care and provide tailored transition care for adolescents living with HIV.

## Introduction

According to recent estimates, 360,582 South African adolescents aged 10–19 years were living with HIV in 2020 [1]. Most ALHIV will require transition from pediatric to adult-based care to ensure continuity of care. ALHIV in South Africa who transition to adult care have suboptimal rates of retention in care and many never record a visit in the adult clinic[2–4]

Poor transition planning can lead to noncompliance with ART, and loss to follow up. Studies have reported disengagement from care during transition, as well as low retention and viral suppression rates after transition [5,6]. In a study in South Africa evaluating retention in care before and after transition to adult care, the retention rate among adolescents who transitioned to adult clinics was 49% compared to 92% among adolescents remaining in pediatric care [5].

Adolescents often lack disease self-management skills and proper preparation before transition. In addition, healthcare providers are unaware of the optimal timing for healthcare transition. Consequently, clinicians make decisions about transitioning adolescents based on their age rather than their individual transition readiness [7,8]. Lack of evidence-based clinical guidance on transition, lack of preparation and the reliance on age for transition, contribute to loss to follow up, virological failure and deaths among ALHIV post transition to adult care [2,3,9,10].

A well-planned transition allows ALHIV to optimize their health, manage their disease independently and assume adult roles and function on issues related to their health [11,12]. Effective transition planning from pediatric to adult-based care is needed to improve engagement in care and the health outcomes. Transition readiness assessments are used in other settings for adolescents with other chronic medical conditions like sickle cell disease, cystic fibrosis, cancers, and chronic renal disease to assist with the timing of transition to adult care. A practical transition readiness assessment tool is needed to guide clinicians in the decision-making process of identifying whether an ALHIV is ready to transition to adult HIV care. The HIV Adolescent Readiness Transition Scale (HARTS) is a validated transition readiness assessment tool recently developed for use among ALHIV in South Africa [13]. Parameters from the HARTS were combined with factors associated with successful transition among ALHIV to develop a transition readiness score [14]. The transition risk score was then embedded in a mHealth app called eHARTS. The goal of eHARTS is to aid clinicians in determining transition readiness and identifying modifiable factors that can help prepare ALHIV for transition.

Mobile health (mHealth) interventions including the use of cell phones, tablets, and personal assisted devices are becoming more common in health-related fields. They provide an attractive alternative to overcome certain barriers in the prevention and management of chronic diseases, including stigma awareness, transportation, clinic costs, and health education [15]. In HIV care, mHealth technologies are used to improve medication adherence, retention in care and viral suppression, as well as address disparities affecting ALHIV in particular along the HIV continuum of care [16,17]. A systematic review identified several types of mHealth interventions that engaged adolescents and young adults living with HIV along the HIV continuum of care in low- and middle-income countries (LMIC), including prevention, diagnosis and linkage to care, adherence and retention, and viral suppression [16]. However, the review did not identify any health interventions addressing healthcare transition for youths living with HIV in LMICs [16]. mHealth interventions are well adapted for youths living with HIV as they commonly engage with social media and mobile health technology. Mobile platforms are also frequently used in health care settings to facilitate care delivery and standardize clinical practice. Our objective was to evaluate the perceived acceptability and feasibility of eHARTS among healthcare providers and ALHIV preparing to transition from pediatric to adult care.

## Methods

### Study design

We conducted a qualitative study to determine the perceived feasibility and acceptability of eHARTS as a transition readiness assessment tool for ALHIV. In this paper, we adhered to the COREQ (COnsolidated criteria for REporting Qualitative research) Checklist in reporting our qualitative study (S1 Appendix) [18]. A total of 30 interviews were conducted with adolescents, clinicians, nurses, counselors, and social workers receiving or providing care in the above clinics. We chose in-depth interviews over focus group discussions because we wanted to demonstrate the app to each participant individually and solicit feedback on eHARTS’s usefulness in transition readiness assessments.

### Study setting

The study took place in Mahatma Gandhi Memorial Hospital, KwaMashu Poly Clinic, and King Edward Hospital, located in KwaZulu-Natal province in South Africa. All are public hospitals and in total, provide care to more than 2,000 children living with perinatally-acquired HIV. As standard of care at each site, adolescents are typically transitioned to adult care between the ages of 15 and 19. According to data from the national antenatal sentinel survey, KwaZulu-Natal had the highest HIV prevalence (41.1%) and an estimated 25% of youths living with HIV aged 15–24 years lived in KwaZulu Natal in 2017 [19].

### Study population and sampling

We utilized a convenience sampling method to recruit adolescents (n = 15) who were receiving HIV care and healthcare providers (n = 15) providing HIV care in Mahatma Gandhi Memorial Hospital, KwaMashu Poly Clinic, or King Edward Hospital from January to March 2022. We purposively recruited middle aged adolescents 14 to 19 as proposed by Kinghorn et al [20]. who were within the potential age of transition. Participants had perinatally acquired HIV and were on ART for at least 6 months and fully aware of their HIV status. Additionally, we included healthcare providers including adult and pediatric clinicians, counselors, nurses, and psychologists who provided care for ALHIV in the aforementioned clinics before or after they transition to adult care. We excluded participants who were unable to read and/or speak English or isiZulu or who were too mentally or physically unwell to provide informed consent. We chose to evaluate potential acceptability, feasibility, and gather information on suggested changes in English before translating to other languages. Adolescents and healthcare practitioners participated in separate interviews.

### Consent and participant Recruitment

HIV care clinicians from the three clinics referred eligible adolescents to participate in the study after each clinic visit. A research assistant then recruited study participants in person. We also included healthcare providers who were caring for ALHIV before or after transition to adult care. Unaccompanied adolescents aged 15 to 18 were offered participation in the study after they signed a written assent form. Following their assent, their caregivers were called by phone to gain verbal consent using a pre-written telephone consent script. Written consent from the primary caregiver was obtained at the next clinic visit. A trained research assistant fluent in English and isiZulu secured written consent from their primary caregivers in English or Zulu, as well as assent from accompanied adolescents under the age of 18. For all adolescents under the age of 15, written parental consent was obtained. Adolescents aged 18 and above and healthcare practitioners gave their own consent. The consenting process took place in a private room. All participants agreed to participate, and efforts were made to recruit an equal number of male and female participants.

### Development of eHARTS

**Fig 1:**
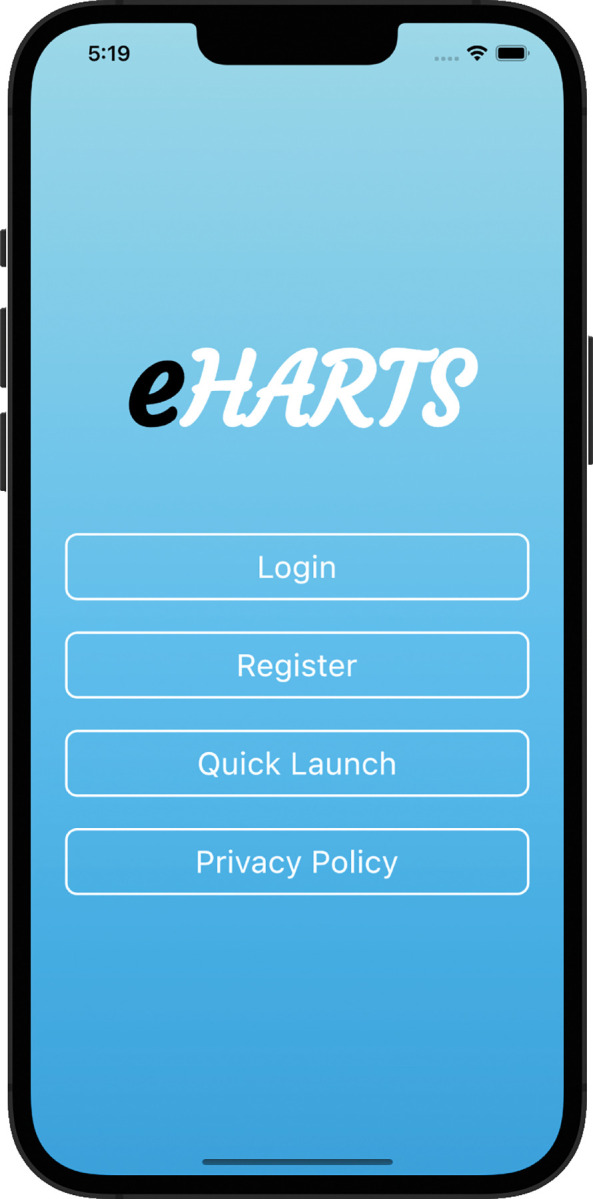
Login page.

**Fig 2:**
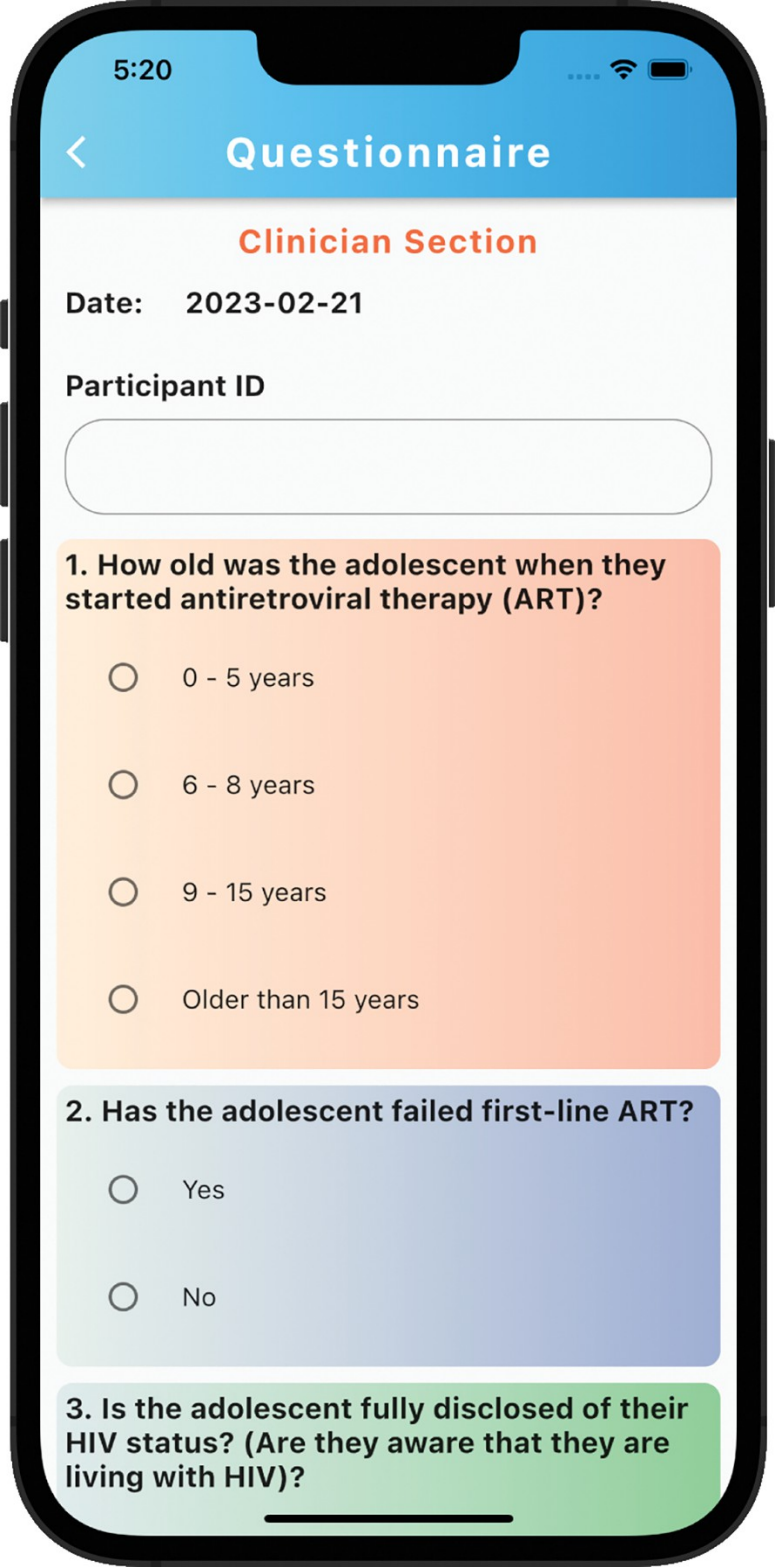
Questionnaire screen.

**Fig 3:**
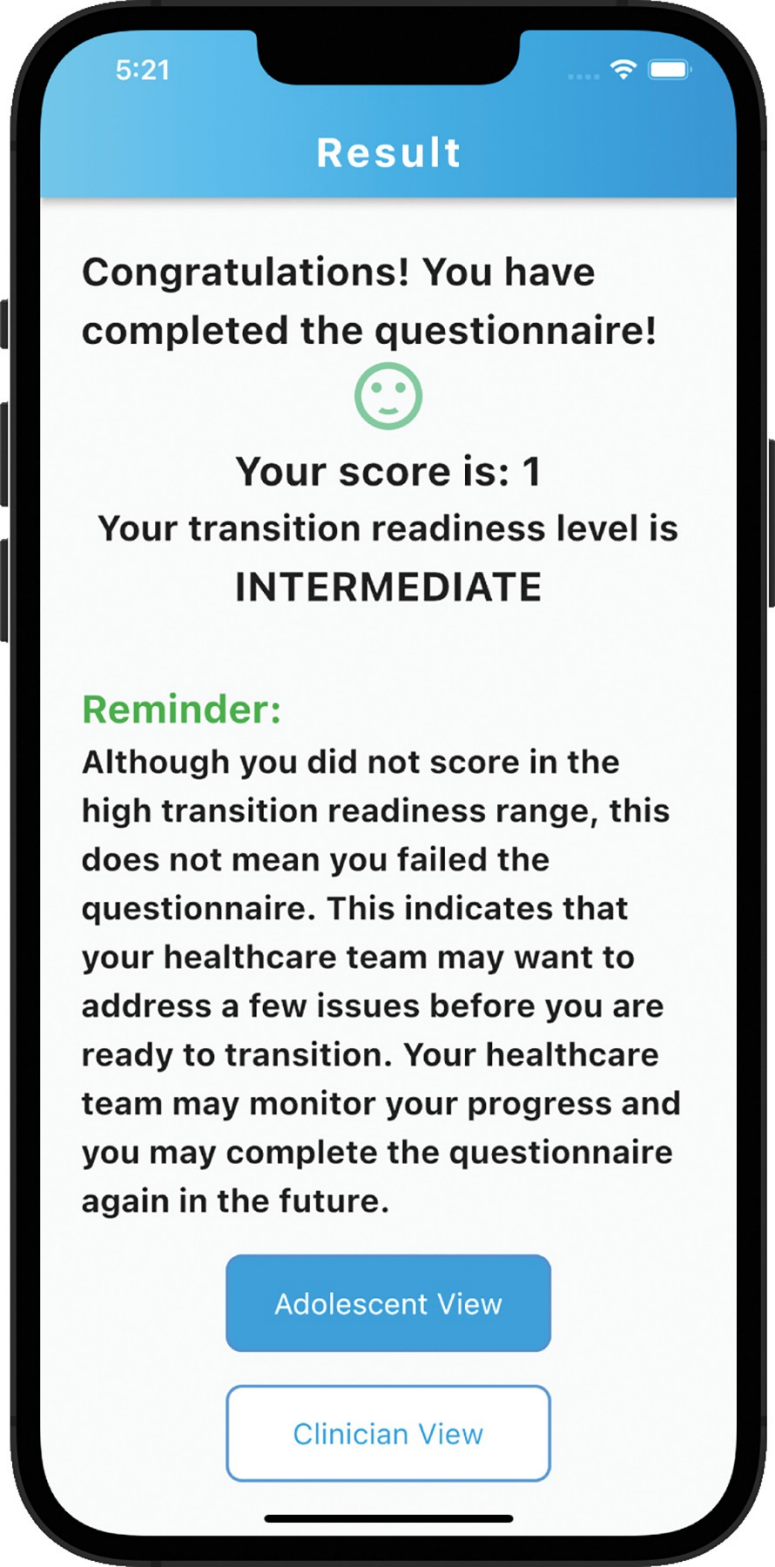
Clinician view of result screen.

**Fig 4:**
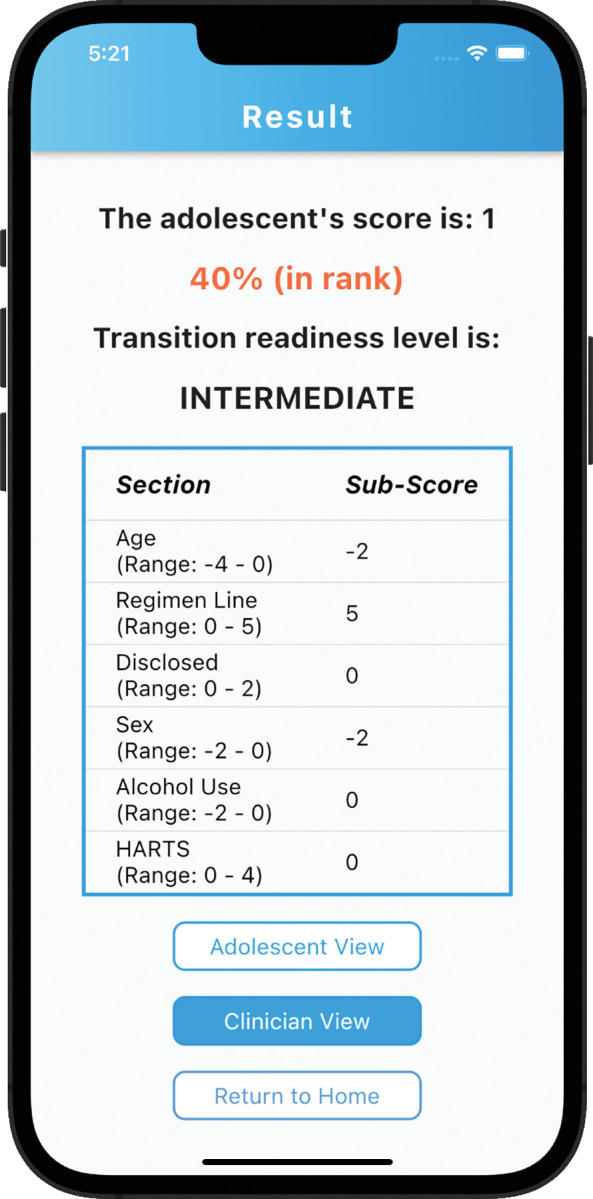
Adolescents view of result screen.

eHARTS is a mobile application used to conduct transition readiness assessments for ALHIV. This version of the eHARTS app is based on formative data from the research group. It combines data from the HARTS questionnaire and other demographic factors that predict successful transition among ALHIV to calculate a transition readiness score [13,14]. The eHARTS app was developed using Flutter and Dart packages and is hosted on Emory University’s Amazon Web Services (AWS) platform. Within the app users answer questions about transition readiness and receive a transition readiness score along with areas that may need improvement prior to transition. The eHARTS questionnaire has a total of 23 questions and is divided into two sections. The first section includes questions for the healthcare provider, while the second section is for adolescents (Fig 1 and Fig 2). The adolescents’ section contains questions on medical knowledge, HIV disclosure, self-efficacy, and healthcare navigation. The eHARTS app then calculates a transition readiness score using a point scoring system, with the final transition score ranging from -8 to 11 and organizes the scores into low, intermediate and high transition readiness based on likeliness of viral suppression after transition to adult care (Fig 3 and Fig 4). Participants who receive a score of 5 or higher are considered to be highly transition ready, and they have the best chance of achieving viral suppression one year after transitioning to adult care. Those with a score of -1 to 4 are in the intermediate transition readiness group, while those with a score -2 or lower are in the low transition readiness category and have the lowest odds of achieving viral suppression after transition. eHARTS will notify the healthcare team of the transition readiness category as well as highlight areas that may need improvement prior to transition to adult care. It can also alert healthcare providers to potential interventions to help prepare adolescents for transition to adult care. Based on the scores, healthcare providers can potentially determine whether an adolescent is ready to transition to adult care and to identify adolescents who need more preparation prior to transition in order to improve their clinical outcomes post-transition. This pilot version of the app was designed and administered in English.

### Data collection

Each interview started with a demonstration of the prototype version of the eHARTS app to participants. After each participant engaged with the app, we conducted in-depth, in-person, semi-structured interviews to obtain relevant information on the perceived acceptability and feasibility of the eHARTS based on three constructs of the Unified Theory of Acceptance and Use of Technology (UTAUT) model of technology acceptance (S2 Appendix) [21]. Our research team developed, piloted, and iteratively refined the interview guide. Adolescents and healthcare providers used the same interview guide to explore relevant topics on the perceived usefulness (performance expectancy), perceived practicality and functionality (effort expectancy), and facilitating conditions, as well as concerns and suggestions on how to improve the app.

Interviews took place in a private room in one of the three clinics. Two female multi-lingual bachelor’s level South African research assistants who are not affiliated with the clinic performed all the interviews (TS and NN). Both interviewers have extensive experience conducting in-depth interviews with ALHIV in South Africa. The interviews lasted 30–60 mins and were conducted in the participant’s preferred language, either English or Zulu. For each interview, one interviewer conducted and audiotaped the interview while the other took field notes. Interviews were transcribed verbatim and, when necessary, translated into English. A research assistant not present during the interviews transcribed and translated the audio recorded interviews. The principal interviewer checked each transcript for quality and correctness.

### Data management and analysis

All personally identifiable information was removed after transcription, and the transcripts were saved on an encrypted, password-protected computer. We used Dedoose software (version 7.0.23, LA, California) to organize and code transcripts, as well as evaluate different themes.

We used an inductive and deductive thematic analysis approach to develop themes tailored to performance expectancy, effort expectancy, and facilitating conditions derived from reviewing, coding, and interpreting the data. Two members of the research team (MF and JN) created inductive codes based on themes that naturally emerged from the data. These team members assigned operational definitions to each code and created a codebook containing illustrative codes by categorizing themes related to performance expectancy, effort expectancy, and facilitating conditions. Any discrepancies were discussed among the team and resolved by consensus for the development of the final codebook. We used Dedoose software to analyze the remaining transcripts applying descriptive codes that addressed the concept of interest and adding new inductive codes to the codebook as new themes emerge. The process was repeated until saturation reached, at which point no new themes or codes were discovered. Author BZ double-checked the coding for consistency across the dataset and accurate application of codes per the code definitions, and any additional conflicts were resolved by consensus among the three coders.

Subthemes were generated to describe different data patterns and category construction about the initial perceived acceptability and feasibility of eHARTS. Major themes relating to the UTAUT model were developed by grouping similar subthemes that reflected representativeness and significant perspectives in the data and addressing the concepts of interest—performance expectancy, effort expectancy, and facilitating conditions. Subthemes were generated to describe different data patterns and category construction about the initial perceived acceptability and feasibility of eHARTS. Major themes relating to the UTAUT model were developed by grouping similar subthemes that reflected representativeness and significant perspectives in the data and addressing the concepts of interest—performance expectancy, effort expectancy, and facilitating conditions. Since this was a pilot demonstration, we did not capture information on social conditions. We captured additional themes on suggested changes and proposed solutions separately. The elaboration and definition for those illustrative codes were categorized based on particular themes.

### Ethical considerations

The Institutional Review Board of Emory University, the Biomedical Research Ethics Committee of the University of Kwazulu-Natal and the KwaZulu-Natal Department of Health approved this study.

## Results

**Fig 5:**
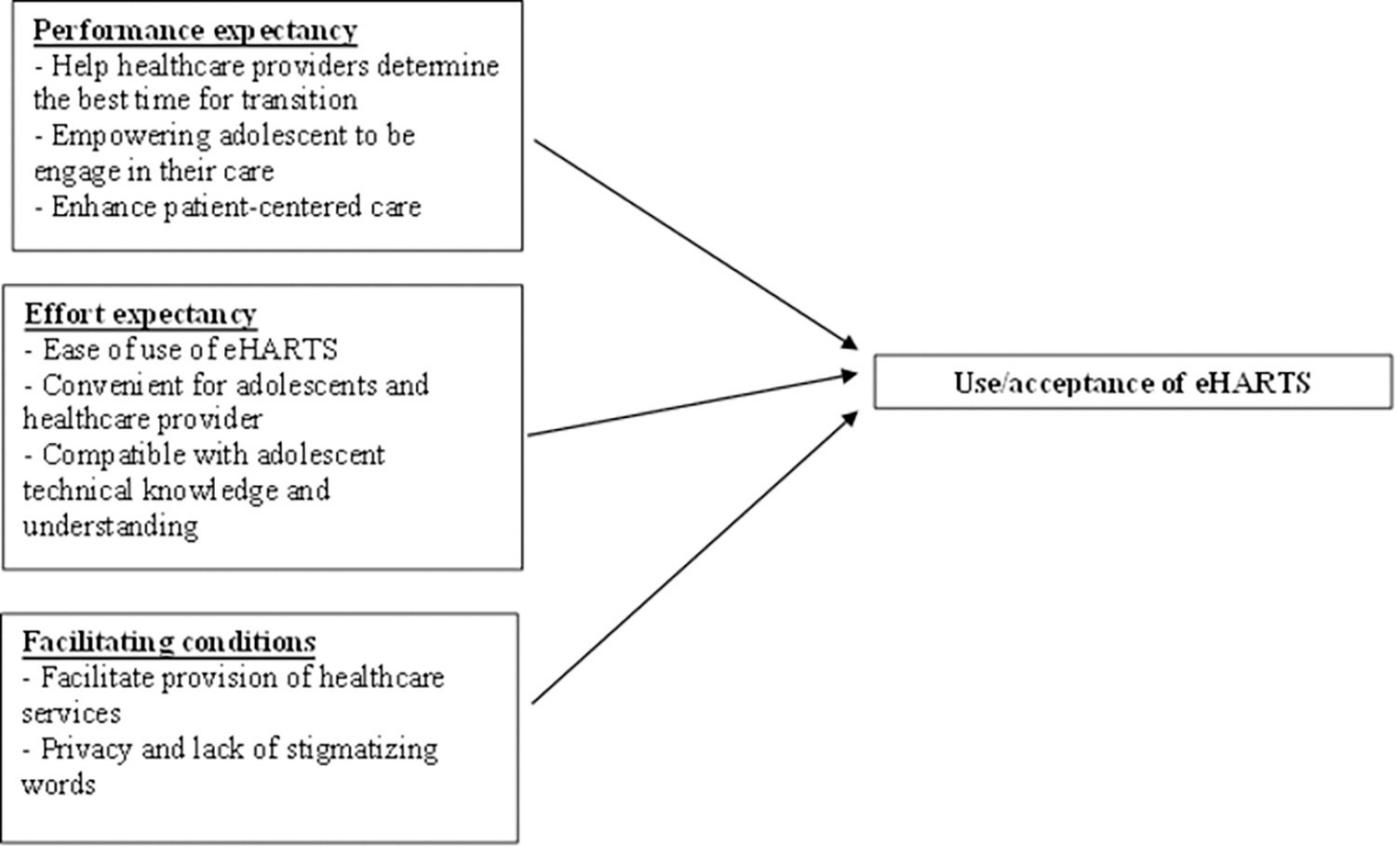
Organization of themes on the perceived acceptability and feasibility following the UTAUT model.

We interviewed 15 adolescents and 15 health care providers. The mean age was 16.2 (standard deviation (SD) 1.2) and 45.2 (SD 11.2) for adolescents and health care providers respectively (Table 1). Most adolescents were male (60%) while most healthcare providers were female (73.3%). The intervention perceived acceptability and feasibility are presented following the UTUAT model and details the performance expectancy, effort expectancy and facilitating condition (Fig 5).

**Table 1:** Descriptive characteristics of study participants (n = 30).

Variable	N (%) or Mean (SD)
Adolescents (N = 15)	
Age (years)	16.2 (1.2)
Female	6 (40%)
Male	9 (60%)
Healthcare providers (N = 15)	
Age (years)	45.2 (11.2)
Female	11 (73.3%)
Male	4 (26.7%)
Doctors	3 (20%)
Counsellors	4 (26.7%)
Nurse	5 (33.3%)
Social Worker	3 (20%)

### Performance expectancy

Participants believed eHARTS was useful in determining the best time to conduct the transition readiness assessment and eHARTS results will assist providers to identify areas that have yet to be addressed.

*“It would even just be useful to identify the areas where the kids are not mature enough in*. *Even if you’re planning to keep the child*, *it gives you an idea of what issues you have not addressed yet*. *So*, *in that sense*, *it is very nice*.*”*–*Physician 2*

Although there was no agreed-upon age at which adolescents should be given the app, participants suggested that eHARTS be given to adolescents aged 10 to 16. However, there were reservations about their literacy abilities as adolescents’ literacy levels may vary, and patients with learning disabilities may have difficulty navigating the app.

“I think that it really depends on the kids, because you may have a 16-year-old who has learning problems, and many of our kids have learning problems. They will find it difficult to navigate through. I think they are quite good, but their language or reading skills might not be very good, and they may be shy to tell you that they are not able to read things…. It’s a bit difficult because some kids will just love doing it, and they will really have fun doing it. But some might not.”- Physician 2

Participants expressed the perceived usefulness of eHARTS both for ALHIV and for healthcare providers. Adolescents, in particular, found the app useful because it gave them the chance to complete it on his own without him being asked questions by someone else. Additionally, it will empower them to ask questions and know more about their health.

“The fact that I am not asked questions by a person, I am reading them on my own and answer them on my own the way I want to respond.”–Adolescent 7

“I like that there are questions that you answer and at the end, it can show you your rate and how do you take your pills. You are also able to talk to a doctor or anyone and ask for help and advise you on how to take care of yourself.”–Adolescents 2

*Healthcare providers also thought of the as empowering for adolescents and it will facilitate their engagement with adolescents* using their responses since many adolescents are reluctant to share their needs with health providers.

“It will empower the adolescents as it asks if they are able to talk to nurses about their different conditions and if they know their viral load. I don’t remember an adolescent asking me about their viral load. They wait for the health care worker to tell them. They will be empowered that they can ask the doctors questions, like doctor my blood was taken last month what are my results? They wait for us to tell them most of the time, I think the tool will be of much help.”–Nurse 2

“…At the same time, it will assist the workers including doctors and nurses because some of the adolescents doesn’t talk. Maybe they don’t talk to me but they are able to speak to a nurse so we will be able to get everything from the app, and also it will help us to not repeat asking same questions because you will know from the app, it will be of much help.”–Counselor 2

The app was reported to enhance patient-centered care by helping healthcare providers identify gaps in adolescents’ care in order to prepare them not only for the transition to adult HIV care but also for the transition from one institution to another.

“Patients might have gaps, which if we do not attend to those issues, the patient might end up defaulting. Some of the questions here, do you understand, do you take your medications alone. If the patient does not take their medications alone, It, could cause some issues for adherence. And if they don’t know why they are taking their medication, they might prefer to stop. So, it is not only helping with transitioning from pediatric to adult, but from institution to institution.”–Physician 3

### Effort expectancy

Both adolescents and healthcare providers found the app navigation features and interface were user friendly.

“It’s easy. It is intuitive to navigate.”–Physician 2

“I like everything about the app, the questions are easy to answer.”–adolescent 4

Healthcare providers also thought the app is well adapted for adolescents very convenient since the questions and answers are straight forward. Although, the very adolescents may require guidance.

“Adolescents can also use it. It is not too hard because it is straight forward questions. There are no paragraph questions here. We only have 1-line questions. The answers are provided, the answers are provided at the end it is straight forward.”–Counselor 1

“They can need guidance, according to their age, they would require someone to assist them” Counselor 4

Adolescents themselves thought the app will be easy to administer and will be well received by their peers because many adolescents are tech-savvy and educated, hence they can complete the app on their own. Adolescents usually have trouble opening to their provider and the app will facilitate engagement with care givers.

“I think adolescents can be able to use it themselves because most people can read now.”–Adolescent 4

“*I am not afraid to answer it*. *Like if I have to be talking to you*, *I get scared and not say other things*.*”–Adolescent 2*

### Facilitating conditions

Given the structure of the HIV treatment centers in South Africa, participants noted that the app could easily be administered to adolescents within hospital premises. Either in the waiting area where the purpose of the app could be explained to groups of adolescents at a time and their individual transition readiness scores made available before they see the doctor, or in the healthcare providers office where they can have one on one conversations.

“Maybe it can be used here the clinic because when they are here, they want to be assisted in every way. It can be properly explained to them and the reasons why it is being done.”–Nurse 4

“While they are waiting, that is the best time. I will do it as a group, and they will be seen individual so that they will be aware when the counsellor administers the app. They should be informed that there will be asked question that prepare them to transition to adult clinic. It is better to prepare them while they are waiting outside, they come in with an idea that they will be asked questions.”–Nurse 1

Even though no specific healthcare provider was designated to administer the app, the availability of various healthcare providers engaged in HIV care for ALHIV makes it possible to any healthcare provider administer the app.

“I think it can be administered by the counselor, the nurse, or during the consultation. I also think it is important that the patient is able to talk to the doctor other than “are there new problems”, “are you sick”, “are you taking your medication”, so I think it can be administered by anyone.”–Physician 3

Other aspect of the app that will facilitate its use in the health facility is its privacy. The app has customized access and unique login details for each user. Additionally, data entering is anonymized and uses only patients’ codes.

“I would say it is very private. Everything is on the phone. The generation of today likes phones, so this app being on the phone will encourage adolescents to do the quiz.”—Adolescent 11

The lack of stigmatizing words would make it easy for adolescents and health care providers to use the app in the health facility.

“I don’t think so, how can they be stigmatized because everyone in this clinic is taking ARVs. I would understand if they were mixed with other adolescents and there is no HIV…There is no HIV mentioned in the app, so I don’t think there will be stigma.”–Nurse 1

Certain challenges were raised that could hinder the use of eHARTS in health facilities. Problems were raised about font size, language, and the false impression eHARTS may give adolescents. A few healthcare providers expressed concern that adolescents may believe the app will be used to transfer them to adult clinics or transfer them to another clinic. This may prevent them from providing honest responses or participating in the transition assessment in general.

“They might want to stay here. They might not want to go to their local clinic where everyone from the community is going. So how do we introduce the app. Not thinking of it as a transitioning tool”—Physician

“It mustn’t be like they are being chase away…Because we are trying to avoid them thinking they are being chased away.”–Nurse

Lastly, a participant thought simply asking the child to complete the quiz without providing feedback for them to assess their level themselves may prevent them from being engaged in their own care:

“These are the areas that maybe you can, you know, because the child does it, now they are not empowered, they just give back the thing and the clinician makes the decision.”–Physician

### Suggested changes and proposed solutions to improve eHARTS

Participants proposed several ways to improve eHARTS and make it more useful for assessing transition readiness for ALHIV in South Africa. The proposed changes included changes to the app’s design, formatting, framing of the application for adolescents, and language.

### App design

Since healthcare providers were concerned that eHARTS might give adolescents the wrong impression. They suggested that the title be framed in such a way that adolescents do not perceive it as a tool for transferring them immediately to adult clinics.

“*And you need to market it in such a way that they do not feel that if they pass today*, *they are going to send me to the other clinic*, *you know*? *Not thinking of it as a transitioning tool… but maybe as a maturity tool or a growing up with HIV tool*. *Rather than the readiness of transitioning*. *I know that is the point*. *But as a clinician*, *it is not just going to help me with transitioning*, *but also how the child is coping with HIV*.*”–Physician 2*

In addition, the purpose of the app should be clearly explained to adolescents, as they will eventually transition at some point. It can be written as an introductory statement on the app and further explained by the person administering the app. A healthcare provider suggested:

“*I think an introduction will be of important you will decide that there will be a brief introduction we do before a person takes a quiz*. *There should be a brief introduction to what is the app is all about*. *Or perhaps a person who will be assisting with administering the app will explain to the adolescent that ‘you are doing this app for these reasons… So*, *it is the same thing with this*, *it like you are doing this app because of A B C and D and we want to achieve A B C and D*. *So*, *the app itself is not aimed to decide to down refer you or not*. *The app aims to access what stage is your health level*. *What resources do you need*, *what support do you need*? *What can be done*? *It is just to give us a picture that what stage are you in as a person*? *It is not to decide for you to be referred*. *You can just leave that part out and just say the app is to make sure that we know your stage of health and what resources you need or support*. *I think it would be better if you put it like that*.*”–Social Worker 3*

### Framing of eHARTS

Participants suggested that eHARTS may be improved by either adding reinforcing messages at the end of the quiz,

“I think for my kids, once you have completed this thing, you want something to pop up and say congratulations, you’ve completed the quiz…It is their score, they should see it and they should be able to decide what they want to make of it before the clinician, or together with the clinician?”–Physician 2

Or the scores can also be presented in a way that is empowering the adolescent:

“…*reminding them if they did not reach a certain score*, *it does not mean they failed*. *It just means that there are gaps on our side as the physicians*. *And if they did not get a good score*, *they can take the quiz again in the future”*–*Physician 3*

Alternatively, a reward system can be used:

“*Maybe telling them if they get a certain score*, *reward them…So I think making a reward*, *if they reach a good score*, *there can be some sort of reward for them*. *They don’t understand how the scoring works so I don’t think they will be tempted to say the wrong thing just to get a higher score*. *So*, *I think that there is a certain reward for them if they reach a certain score*. *And if they don’t*, *they need to understand that it is not their fault*, *it is just a reflection on us as service providers*.*”–Physician 3*

### Language

Participants provided suggestions on how the language should be translated and integrated into the final version of the app. Many participants suggested that the app be translated into isiZulu so that adolescents can choose which language they want to use while still understanding the question and providing valid responses.

“It would be better if you use both languages. Write in isiZulu and include English as well. Other adolescents would read isiZulu and don’t understand but they understand when they are reading in English. Others don’t understate reading in English but when they are reading the same thing in isiZulu they understand.”—Adolescent 4

Other suggestions were to include other local languages target non-Zulu speaker. Some participants suggested that both languages be included in eHARTS:

“*With the language*, *I think the 15-year-old understands English sometimes isiZulu is difficult*, *and we are not targeting Zulu speakers only*, *there are Sotho’s’ as well and many other languages*, *so the language should be right for everyone*.*–Nurse 2*

## Discussion

This study presents a qualitative assessment of the perceived acceptability and feasibility of the eHARTS for conducting transition readiness assessments, as well as opportunities for improving eHARTS for transition assessments. Overall, participants perceived eHARTS to be acceptable and feasible to implement in HIV clinics in South Africa because it was simple to use, non-stigmatizing, and could be implemented in the hospital without additional strain on the clinic. In addition, participants felt that the questions were straightforward, necessitating lesser efforts on the part of adolescents or healthcare providers. Participants also believed eHARTS was useful since it could facilitate communication with adolescents and empower them to be more involved in their own care. Healthcare providers also felt that eHARTS will also be able to prepare ALHIV not only for transition to adult HIV clinics but also to other institutions.

Based on three of the UTAUT constructs: performance expectancy, effort expectancy, and facilitating conditions, eHARTS appears to be acceptable among ALHIV and healthcare providers [22]. Most participants agreed that eHARTS would measure transition readiness for ALHIV in South Africa as expected (performance expectancy) and could be used in the waiting room during routine clinic visits with minimal effort on the part of both adolescents and healthcare providers (effort expectancy). Furthermore, participants believed that the HIV clinic organizational structure in KwaZulu-Natal could facilitate the integration of the app into ordinary clinic activities without influencing wait times at the clinic (facilitating conditions). These constructs are strong determinants of users’ behavioral intentions toward any technology, and they adequately capture participants’ intentions to adopt and subsequently use eHARTS.

This study fills an important gap in the realm of transition readiness assessment for ALHIV. eHARTS, like other transition readiness scales, can be used to assess adolescents’ preparedness to transition to adult care and identify whether they have self-management skills. Existing transition scales are too general to be useful for people living with HIV. The Transition Readiness Assessment Questionnaire (TRAQ), for example, is the most widely used transition readiness assessment tool [23]. This scale is not disease-specific and its usage in developing countries has not been validated. It doesn’t consider the complexities of living with a stigmatizing disease like HIV and the unique barriers to successful transition for ALHIV. Furthermore, the TRAQ is based exclusively on self-report from adolescents and contains no objective measure of whether the adolescents have truly acquired these skills. The UNC TR[x]ANSITION tried to overcome this problem by correlating results with medical data to validate participants’ responses, but it fell short due to the targets being poorly correlated with their respective domains and the increased administrative burden of cross-referencing each domain with medical records [24]. In comparison to these generic tools, eHARTS uses HIV-specific features from the HARTS questionnaire, such as disclosure. It also incorporates the input of healthcare practitioners as well as measures that are highly predictive of successful transition to create a subjective and objective measure of transition preparedness. Moreover, the administration of eHARTS has a lower administrative burden.

Many mHealth initiatives have been developed as a result of the fast adoption of mobile technology to ameliorate HIV care for people living with HIV in low- and middle-income countries. A recent systematic review identified various types of mHealth interventions that engage youths and adolescents living with HIV in low- to middle-income countries along the HIV continuum of care [16]. These interventions varied from simple text messages to more complex interventions involving interactive games and peer engagement. The review found no mHealth intervention addressing transition for adolescents and young people living with HIV. To the best of our knowledge, this is the first mHealth intervention that focuses on transition readiness assessments for ALHIV. An mHealth intervention iTRANSITION, is being developed in the US to facilitate transition of youths living with HIV from pediatric to adult clinics [25]. Although, the study is being conducted in a setting with different healthcare transition models, it could potentially be adapted to other settings in the future. However, results from this study are yet to be published limiting any conclusion on its transferability to LMIC.

While the majority of these mHealth interventions have shown promise in terms of improving health-related outcomes, they face operational challenges and have limited sustainability in resource-constraint settings due to poor network access, rising mobile data cost, low mobile phone ownership among adolescents, and the ongoing need for training the persons in charge of administering the intervention or the participants themselves [26,27]. eHARTS provides numerous advantages in this regard in that it doesn’t require any prior knowledge or training for its use. In addition, adolescents are not expected to own a phone or purchase mobile data. eHARTS could be readily accessible at HIV clinics, where it could be used by adolescents during their visits.

The findings of this study must be viewed in the context of their limitations. The research was conducted with young adolescents who had acquired HIV perinatally. As a result, the findings do not reflect the perspective of adolescents who may have transitioned or those who acquired HIV through behavioral means. However, we were able to obtain findings that may reflect the perspective of those other adolescents by including healthcare practitioners with experience working with adolescents of various age groups and HIV transmission modes.

Another limitation was the sample size. Although the sample size is sufficient to qualitatively elicit participants’ perspectives on the acceptability and feasibility of eHARTS, the results, however, cannot be extrapolated to all ALHIV because of the limited sample size.

Participants mentioned language as a major barrier for eHARTS. Given that this was a pilot test, we demonstrated the app in English for practical reasons. The next phase of the intervention will be to include participant comments, translate the app into other South African local languages, and retest its acceptability and feasibility.

## Conclusion

Our findings indicate that eHARTS is a promising tool for transition readiness assessment for ALHIV in South Africa. This study found that eHARTS has a high level of acceptability and feasibility among adolescents and healthcare providers. Our findings close a significant gap in practice and research on transition readiness assessments for ALHIV. It assessed the electronic delivery of a validated healthcare transition questionnaire that is disease-specific and region-specific. It also highlights the potential effectiveness of conducting transition readiness assessments for ALHIV in HIV care clinics as it can facilitate transition readiness assessments in settings where they are not currently done, helps clinicians provide differentiated transition care for ALHIV and can address individual challenges to transition faced by ALHIV.

## Supporting information

S1 Appendix COREQ (COnsolidated criteria for REporting Qualitative research) Checklist. (DOCX)

S2 Appendix eHARTS Interview Guide. (DOCX)
