# Supplementary material for: A qualitative assessment of the perceived acceptability and feasibility of eHARTS, a mobile application for transition readiness assessment for adolescents living with HIV in South Africa
Source: PLOS Digit Health. 2023 Jun 16;2(6):e0000272. doi: 10.1371/journal.pdig.0000272 (PMC10275417; doi:10.1371/journal.pdig.0000272)
Supplement: S2 Appendix — (DOCX) [file pdig.0000272.s002.docx]

**S2 Appendix: eHARTS Interview Guide**

Questions:

First, let’s talk about the idea of the eHARTS in general.

1. How do you think a transition readiness app would be useful? *Probe on use in healthcare providers and adolescents*
2. What do you like about the eHARTS app? *Probe on data storage and tracking, ease of use.*
3. What don’t you like about the eHART app? *Probe font, scrolling, login, display*
4. What changes would you make to eHARTS?

Second, let’s talk about the eHARTS App.

1. What data should be collected for monitoring transition progress? *Probe information to add or subtract*
2. What should the App look like? *Probe on font, scrolling, arrangement of questions.*

Third, I’d like to know how you’d want to use eHARTS.

1. How would you use the eHARTS App?
2. When should it be used? *ages of adolescents*
3. How should you arrange to use the app in clinic? *Probe use of nurses, counselors, administrators to begin use.*
4. Where should it be used? *Probe in clinic, waiting room, before visit.*

Fourth, I’d like to know how you’d like to get instructions on using the eHARTS App.

1. How should we explain the use of eHARTS? *Probe on pamphlet with cartoons versus photos, use of color, demonstration of App*
2. What language(s) should be used?
3. Who should be shown how to use the eHARTS App? *Probe on healthcare providers only or would adolescents know how to use it*

Finally, please tell me any other thoughts you have about the *eHARTS App.*

1. What are your concerns about the eHARTS App? *Probe on stigma/privacy.*
2. Tell me anything else you’d like about this program. Are there other things we should consider?

Thank you for your participation in this study.
